# Prevalence of prediabetes and associated factors among community members in rural Isingiro district

**DOI:** 10.1186/s12889-023-15802-9

**Published:** 2023-05-25

**Authors:** Isaac Petit Ampeire, Peter Chris Kawugezi, Edgar Mugema Mulogo

**Affiliations:** Mbarara University of Science and Technology, Mbarara, Uganda

**Keywords:** Prediabetes, Prevalance, Associated fators, Rural Isingiro, Rural Uganda population

## Abstract

**Background:**

In rural Uganda a significant number of persons afflicted with pre-diabetes are unaware of the condition. This is likely to lead to diabetic complications resulting in catastrophic health expendirure.The burden of prediabetes in rural Isingiro has not previously been determined. This study examined the prevalence of prediabetes and the associated factors among rural community members.

**Methods:**

We conducted a cross-sectional survey and enrolled 370 participants aged between 18 and 70 years in the Kabuyanda sub-county, rural Isingiro district in march 2021. Multistage sampling and systematic random sampling were conducted to select eligible households. Data was collected using a pretested WHO STEP-wise protocol questionnaire. The primary outcome was prediabetes (FBG = 6.1mmol/l to 6.9mmol/l), calculated as a proportion. Participants known to be diabetic or on medication were excluded. Chi-square tests and multivariate logistic regression model were performed for data analysis using STATA.

**Results:**

The prevalence of prediabetes was 9.19% (95% CI 6.23–12.14). Independent factors significantly associated with pre-diabetes were; advancing age [AOR = 5.7, 95% CI:1.03–32.30], moderate-intensity work [AOR = 2.6,95% CI:1.23–5.63], high level of consumption of a healthy diet [AOR = 5.7, 95% CI:1.67–19.05] and body mass index [AOR = 3.7, 95% CI:1.41–9.20].

**Conclusion:**

Prediabetes is prevalent among adult community members in rural Isingiro, southwestern Uganda. Age and lifestyle factors predict prediabetes in this rural population, suggesting a need for targeted health promotion interventions.

## Background

Diabetes is a chronic condition that occurs when there are raised levels of glucose in the blood because the body cannot produce any or enough of insulin or use insulin effectively [1]. Diabetes is an insidious disease. Patients usually go through the stage of prediabetes for some years before they develop diabetes disease [2]. Diabetes mellitus is a major public health concern of the 21st century [3]. It is one of the four major Non-communicable diseases (NCDs) namely cardiovascular diseases, carcinomas and chronic lung diseases.Dabetes kills and disables, striking people at their most productive age, impoverishing families, and reducing the life expectancy of older people. The burden of diabetes drains national healthcare budgets and slows economic growth. It is a major cause of catastrophic expenditure for vulnerable households.

Global prevalence of diabetes has more than doubled over the past two decades from 4.6% in the year 2000 to 10.5% in 2021. Prevalence is projected to increase to 12.2% by 2045. On the African continent, the prevalence of diabetes is currently at 4.5% but is expected to raise to 4.8% by 2030 [4]. A large proportion of this population remain unaware that they are afflicted by the condition,undiagnosed diabetes has costly public health implications in Africa [5].

Prediabetes is a forerunner to type 2 diabetes [6]. It is an intermediate stage of raised blood glucose between normal glucose tolerance and type 2 diabetes. Prediabetes is also associated with nephropathy, small fiber neuropathy, diabetic retinopathy, and an increased risk of macro vascular disease [7]. The World Health Organization (WHO) defines prediabetes by fasting plasma glycemic levels that are higher than normal but lower than diabetes thresholds, that is, fasting plasma glucose of 6.1mmol/l to 6.9 mmol/l. Prediabetes is also called intermediate hyperglycemia which is defined by two conditions; impaired glucose tolerance (IGT) or impaired fasting glucose (IFG) [4]. The global prevalence of prediabetes based on IFG was estimated at 319 million (6.2%) in 2021 and is projected to increase to 441 million (6.9%) in 2045[4]. According to the International Diabetes Federation (IDF), the African regional prevalence of IGT among adults aged 20–79 was estimated at 40.9 million (8.0%) in 2021 and is projected to increase to 84.7 million which will be more than 50% increase.

Several studies in sub-saharan Africa have found differing prevalence of prediabetes. Analysis of the Namibian demographic and health survey among adults aged 35–64 years in 2013 found a prediabetes prevalence of 6.8% [8]. In a cross-sectional survey in a rural community in Nigeria among individuals aged above 15 years in 2015, the prevalence of prediabetes was 2.4% [9]. In the same year in the national risk factor survey on NCDs conducted in Kenya, the prevalence of prediabetes was 3.1% among adults aged 18 years to 69 years [10].In Cameroon, a systematic review and meta-analysis of cross-sectional studies that included healthy adults published between 2000 and 2017, found a prevalence of prediabetes at 7.1% [11].In Uganda, the first and only documented population-based risk factor survey on NCDs in adults aged 18–69 years was conducted in 2014. This survey found an overall prevalence of IFG at 2.0% [12]. However, previously in 2013 another study conducted among adults aged 30–60 years in eastern Uganda, found a prevalence of IFG at 8.6% [13]. Early screening for prediabetes identifies an important target group for interventions aimed at preventing diabetes and its debilitating complications. The burden of pre-diabetes in rural Isingiro had not previously been determined. This study aimed to examine the prevalence of pre-diabetes and the associated factors in Isingiro.

## Methods

### Design and study setting

A cross sectional study using quantitative data collection was used. It was conducted in Kabuyanda sub-county, in Isingiro district one of the districts in south western Uganda region, between September 2020 and March 2021. The sub-county has 7,864 households with a total population of 37,569 of which 19,722 are female. The sub-county is comprised of 8 parishes with 60 villages. Most of the community members derive their livelihood from subsistence farming.

### Sample size and sampling procedure

Multistage cluster sampling was used to select the villages (clusters) to be included in the study. One half of the total parishes in the sub-county were randomly selected. The villages were randomly selected from each parish based on probability proportional to the size sampling. The list of households in the selected village clusters were obtained and used as a sampling frame. A sampling interval was calculated and systematic random sampling was then done. The number of households from each village was selected randomly based on probability proportional to the size sampling. In each household, participants aged 18 years and above, and not more than seventy years, were selected with one participant chosen by lots per household. The sample size estimate was based on a formula for cross-sectional surveys. A “p” prevalence of pre-diabetes of 13.8% was assumed. A sample size of 370 was calculated.

### Data collection instruments and process

Data was collected by trained nurses using a structured pretested questionnaire adapted from the WHO STEP-wise approach for risk factors surveillance of Non-communicable diseases. Participants that reported having diabetes or on medication for diabetes were excluded. A standing height was measured to the nearest 0.1 cm using a portable stadiometer (Fazzini S225) and recorded as the maximum distance from the floor base of the stadiometer to the highest part on the head, with the participant standing barefooted in a fully extended standing position. Weight was measured using a portable well-calibrated mechanical weighing scale (Fazzini S758) to the nearest 0.1 kg and adjusted to zero before each reading.

The body mass index (BMI) was calculated as weight in kilograms over height in meters squared and was categorized as normal (BMI < 25 kg/m^2^), overweight (25 kg/m^2^ ≤ BMI < 30 kg/m^2^), and obesity (BMI ≥ 30 kg/m^2^). Waist circumference was measured using a constant tension tape placed on the skin of the waist across the mid-point between the lower margin of the least palpable rib and the top of the iliac crest with the participant standing with feet close together, arm at the side and back straight. Blood pressure measurements were taken according to the European society of cardiology and hypertension 2018 guidelines for the management of hypertension [14].

Participants were requested to have their last meal at a time not more than 10:00 pm in the night to achieve 8 h of fasting. Fasting blood glucose was tested by taking participants’ capillary blood sample from the ring finger of the non-dominant hand according to the WHO guidelines on drawing blood 2010[15] .The level of blood glucose was measured using a validated digital glucometer (Free style optium neo, Abbott) and recorded in mmol/l. Prediabetes was defined as fasting blood glucose of 6.1mmol/l to < 7mmol/l.

Physical activity data was obtained from the participant self-report responses to the standardized questions for physical activity adapted from the WHO STEPs instrument. Physical activity was categorized according to the levels of activity reported by the participants based on the standard categories from WHO recommendations on physical activity in adults as; vigorous intensity work, moderate intensity work, active transport, vigorous recreation and moderate recreation [16]. Data on dietary patterns were obtained based on self-reports for the frequency of consumption of foods categorized as healthy or diabetogenic over a week. The frequency groups were defined by how often a food was eaten in week. These were ; none, once a week, twice a week or more than 3 times a week. The dietary patterns were then categorized as low (none and once a week), medium (twice a week) or high (more than 3 times a week). Diabetogenic foods included; meat, milk, sugary beverages, factory cooking oil and bolted grain flour breads. A healthy diet included; fruits, vegetables, whole grains, nuts and seeds as defined by WHO and the international diabetes federation (IDF) criteria.

### Data analysis

Data was entered into Access to create a data base that was transferred to Stata version 12 (College Station, Tx 77,845) for analysis. Continuous variables were expressed as means and standard deviation while categorical variables were expressed as percentages. The estimated odds ratio was reported with a 95% level of confidence and a p-value less than 0.05 as a cut off point for statistical significance. Fasting blood glucose levels were coded with a 0 for blood glucose ≤ 6mmol/l and 1 for blood glucose of 6.1 to < 7mmol/l, prediabetes was then recoded if the code was 1 and its prevalence computed as a proportion. Bivariate analysis was perfomed to assess the association between explanatory variables, and prediabetes. Logistic regression was performed to determine variables that were independently associated with prediabetes.

This study was approved by the Mbarara University of science and technology faculty of medicine research committee (FRC) and the Mbarara University of science and technology research ethics committee (MUST-REC).

## Results

### Prevalence of prediabetes

A total of 378 eligible participants were contacted, out of which 370 (97.8%) were enrolled and participated in the study. A total of 5 participants missed in the early morning of day two for testing for blood sugar and 3 failed to achieve fasting status in the morning.

Out of 370 participants, 34 had prediabetes with a prevalence of 9.2% (95% CI:6.23–12.14), while 336 (90.8%) did not have prediabetes (Table 1).

**Table 1:** Prevalence of prediabetes

Status	n (%)
**Prediabetes**	34 (9.2)
**Normal**	336 (90.8)
**Total**	370 (100)

### Social demographic characteristics of the participants

Of the 370 participants 119 (32.2% were males and 251(67.8%) were females. The overall mean age was 42 (SD = 14.3). While the mean age for males was 41 (SD = 13.7), for females it was 42 (SD = 14.5). More than half of the participants had primary level education, family history of diabetes was reported in only 23.4% of the participants. Among the social demographic factors, age and level of education had a significant association with prediabetes (p < 0.05) at the chi-square test analysis (Table 2).

**Table 2:** Socio-demographic characteristics of the respondents

Characteristic	Total (N = 370) n (%)	No prediabetes (N = 336) n (%)	Prediabetes N = 34 n (%)	P-value
**Age (mean, SD)**	42, SD = 14.3			
**Age groups**				0.012**
18–30	93 (25.1)	91 (27.2)	2 (5.9)	
31–45	123 (33.2)	113 (33.6)	10 (29.4)	
46–60	111 (30.1)	94 (27.9)	17 (50.0)	
61–70	43 (11.6)	38 (11.3)	5 (14.7)	
**Gender**				0.129
Male	119 (32.2)	112 (33.3)	7 (20.6)	
Female	251 (67.8)	224 (66.7)	27 (79.4)	
**Marital status**				0.767
Married/Cohabiting	285 (77.2)	259 (77.1 )	26 (76.5)	
Separated/Divorced	23 (6.2)	21 (6.3)	2 (5.9)	
Widowed	38 (10.3)	33 (9.8)	5 (14.7)	
Never Married	24 (6.3)	23 (6.9)	1 (2.9)	
**Level of education**				0.018**
No formal education	78 (21.1)	69 (20.5)	9 (26.5)	
Primary level completed	207 (56.0)	191 (56.8)	16 (47.0)	
Secondary level completed	57 (15.4)	54 (16.1)	3 (8.8)	
College /university completed	17 (4.6)	15 (4.5)	2 (5.9)	
Refused	11 (3.0)	7 (2.1)	4 (11.8)	
**Monthly income (UGX)**				0.069
<50,000	218 (58.9)	198 (58.9)	20 (58.8)	
50,000-100,000	113 (30.5)	99 (29.5)	14 (41.2)	
100,000-500,000	39 (10.5)	39 (11.6)	0	
**Employment**				0.378
Self Employed	346 (93.5)	313 (93.2)	23 (67.7)	
No formal employment	24 (6.5)	23 (6.9)	1 (2.9)	
**Family history of diabetes**				0.084
Yes	86 (23.3)	81 (24.1)	5 (14.7)	
No	259 (70.0)	230 (68.5)	29 (85.3)	
Don’t know	25 (6.8)	25 (7.4)	0	

** Statistically significant at p < 0.05

### Association between variables and prediabetes at bivariate analysis

#### Social demographic variables

Advancing age was significantly associated with prediabetes. The analysis revealed that the odds of prediabetes were 8 times higher with increasing age (COR = 8.2 95% CI: 1.84–36.70), age was therefore included in the multivariate analysis. Other social demographic factors, that is, education level, household income, and family history of diabetes did not show significant association with prediabetes and were therefore not included in the multivariate analysis model (Table 3).

### Lifestyle variables

Among the lifestyle variables, increasing body mass index, hypertension, moderate intensity level of work, and healthy diet were significantly associated with prediabetes. Participants with higher BMI were 3 times more likely to have prediabetes (COR = 3.8, 95% CI:1.58–8.94).The odds of prediabetes were twofold among those who were hypertensive (COR = 2.1, 95% CI:1.05–4.35). Participants with a moderate intensity level of work were twice likely to have prediabetes (COR = 2.5,95% CI:1.25–5.24). All the other four categories of physical activity were not significantly associated with prediabetes and therefore not reported. The odds of prediabetes were 5 times higher among participants who consumed a high level of food categorized as healthy (COR = 5.8,95% CI:1.79–18.95) compared to those who consumed them at a low level (Table 3).

Smoking and alcohol use were not significantly associated with prediabetes, so they were not included in the multivariate analysis model. Increasing BMI, hypertension, moderate intensity level of work and healthy diet were included in the multivariate analysis.

### Medical and health access variables

Bivariate analysis of medical and health access variables showed that ever testing for diabetes and steroid use were significantly associated with prediabetes. The analysis revealed that the odds of prediabetes were twice as high among those who had ever tested for diabetes (COR = 2.6, 95% CI:1.02–6.33). The participants who used steroids were twice as likely to have prediabetes (COR = 2.1,95% CI:1.02–4.33) compared to those who did not use steroids (Table 3).

Ever testing for diabetes and history of steroid use were therefore included in the multivariate analysis model.Availability of testing services at the health facility obtained from participant reports was not significantly associated with prediabetes and was thus not included in the multivariate analysis model.

**Table 3:** Association between variables with prediabetes at bivariate analysis

Variable		Prediabetes n (%)		COR	95% CI	P-value
		No Prediabetes N = 336	Has Prediabetes N = 34			
**Age**	18–30	91 (27.1)	2 (5.9)	1.0*		
	31–45	113 (33.6)	10 (29.4)	4.0	0.85–18.87	0.077
	46–60	94 (27.9)	17 (50.0)	8.2	1.84–36.70	0.006**
	61–70	38 (11.3)	5 (14.7)	5.9	1.11–32.29	0.037**
**Education level**	No formal education	69 (20.9)	9 (30.0)	1.0*		
	Primary level	191 (58.1)	16 (53.3)	0.6	0.27–1.52	0.314
	Secondary level	54 (16.4)	3 (10.0)	0.4	0.11–1.65	0.217
	college /university	15 (4.6)	2 (6.7)	1.0	0.20–5.22	0.979
	*Refused*	*11 respondents declined to mention their level of education.*				
**Household income**	< 50,000	198 (58.9)	20 (58.8)	1.0*		
	50,000–100,000	99 (29.5)	14 (41.2)	1.4	0.67–2.88	0.363
	100,000–500,000	39 (11.6)	0	1.0		
**Family history of diabetes**	No	230 ( 73.9)	29 (85.3)	1.0*		
	Yes	81 ( 26.1)	5 (14.7)	2.0	0.76–5.45	0.154
**Smoking**	No	288 (85.7)	25 (73.5)	1.0*		
	Yes	48 (14.3)	9 (26.5)	2.2	0.95–4.90	0.066
**Alcohol use**	No	186 (55.4)	19 (55.9)	1.0*		
	Yes	150 (44.6)	15 (44.1)	0.9	0.48–1.99	0.953
**Body mass index**	Normal	217 (64.6)	16 (47.1)	1.0		
	Overweight	83 (24.7)	8 (23.5)	1.3	0.53–3.16	0.553
	Obese	36 (10.7)	10 (29.4)	3.8	1.58–8.94	0.003**
**Hypertension**	Normal	229 (68.2)	17 (50.0)	1.0*		
	High	107 (31.9)	17 (50.0)	2.1	1.05–4.35	0.036**
**Moderate intensity work.**	No	225 (66.9)	15 (44.1)	1.0*		
	Yes	111 (33.0)	19 (55.9)	2.6	1.25–5.24	0.010**
**Dietary Patterns**						
	Low	105 (31.3)	4 (11.8)	1.0*		
Healthy diet	Medium	177 (52.7)	18 (52.9)	2.7	0.87–8.10	0.083
	High	54 (16.1)	12 (35.3)	5.8	1.79–18.95	0.003**
**Ever tested for diabetes**	No	305 (91.9)	27 (8.1)	1.0		
	Yes	31 (81.6)	7 (18.4)	2.6	1.02–6.33	0.044^**^
**Reported testing services at health facility**	No	251 (91.3 )	24 ( 8.7)	1.0		
	Yes	59 (85.5)	10 (14.5)	1.8	0.80–3.90	0.156
	Don’t know	26 (100.0)	0	1.0		
**Steroid use**	No	190 (56.6)	13 (38.2)	1.0*		
	Yes	146 (43.5)	21 (61.8)	2.1	1.02–4.33	0.044**

** Statistically significant at p < 0.05

1.000*- odds ratio of the referent category

### Factors associated with prediabetes

From the multivariate analysis of the significant variables identified at bivariate analysis, increasing age, moderate intensity level of work, consumption of high level of diet categorized as healthy and BMI remained significantly associated with prediabetes with p values < 0.05. We adjusted for confounding factors based on biological relevance and significant associations found in the bivariate analysis. The final model was adjusted for family history of diabetes, smoking, hypertension, ever testing for diabetes and use of steroids.

The multivariate analysis showed that the odds of prediabetes were 5.7 higher with increasing age (AOR 5.7,95% CI:1.03–32.30). The odds of prediabetes remained twice as much for participants who had moderate intensity work (AOR = 2.6,95% CI:1.23–5.63). Participants who consumed a diet categorized as healthy at high levels were 5 times more likely to have prediabetes (AOR = 5.7,95% CI:1.67–19.05). The Odds of prediabetes were 3.6 times higher among the participants with a higher BMI (AOR = 3.7, 95% CI:1.41–9.20)(Table 4).

**Table 4:** Multivariate analysis results of factors associated with prediabetes

Characteristic		COR	P value	AOR	P value	95% CI
Age	18–30	1.0				
	31–45	4.0	0.077	3.5	0.11	0.73–17.03
	46–60	8.1	0.006	7.4	0.01	1.61–34.29
	61–70	5.9	0.037	5.7	0.046^**^	1.03–32.30
Moderate intensity work	No	1.0				
	Yes	2.5	0.010	2.6	0.012^**^	1.23–5.63
	Low	1.0				
Healthy diet	Medium	2.7	0.083	2.4	0.13	0.77–7.60
	High	5.8	0.003	5.7	0.005^**^	1.67–19.05
	Normal	1.0				
BMI	overweight	1.3	0.553	1.0	0.918	0.41–2.64
	obese	3.8	0.003	3.7	0.007^**^	1.41–9.20

** Statistically significant at p < 0.05

## Discussion

The study findings show an overall prevalence of prediabetes of 9.2%. This was considerably higher than the estimated global prevalence but only slightly higher than that of Africa region alone [4]. In contrast, another study that assessed the prevalence of Prediabetes based on correlation with HbA1c in Sub-Saharan Africa found a prevalence of 25% by the American Diabetes Association (ADA) criteria [17]. The ADA estimates a higher prevalence of prediabetes than the WHO criteria that we used in the current study. We found a slightly higher prevalence of prediabetes compared to what Mayega et al. found in rural eastern Uganda 7 years ago. The prevalence of prediabetes in rural eastern Uganda was 8.6% by the same WHO criteria among adults aged 35 to 60 years [13].

The study findings also reveal that the prevalence of prediabetes was higher among women (10.7%) compared to men. Systematic reviews of community-based cross-sectional studies in sub-saharan Africa that provided sex- specific prevalence, demonstrated a higher prevalence of prediabetes in males than in females by the IFG, but higher prevalence in women by IGT [18]. A study conducted in Kenya among hypertensive individuals aged above 18years, found a higher prevalence of prediabetes among females (3.3%) compared to males (2.8%) [19], this difference by sex is similar to our study findings though we included non-hypertensive respondents. The prevalence of prediabetes by sex varies with regions. We found that women in this region have moderate intensity level of work and no vigorous work or physical exercise, they are also relatively more overweight and obese compared to the males. This could explain why they have a higher prevalence of prediabetes.

The prevalence of prediabetes was highest among the age group of 46 to 69 years. This is similar to other studies that showed that prediabetes is more prevalent in the age group of 30–69 years [2] [20]. Our study has shown that prediabetes is prevalent in this predominantly rural region of Uganda and is comparable with regional estimates to require considerable attention to address it as a stage of prevention for type 2 diabetes.

Analysis of the social demographic factors, showed that advancing age was significantly associated with prediabetes. The odds of prediabetes were fivefold with advancing age. We found that 1 in 6 adults aged more than 45 years may have prediabetes. This finding confirms that advancing age remains a significant risk factor for prediabetes in this rural population. Advancing age is a well-known independent risk factor for insulin resistance. However, we also found that most of the adults in this region do not engage in any form of vigorous or even moderate intensity exercises. The high level of physical inactivity and increased risk for insulin resistance could be the reason for the significant association of advancing age with prediabetes in our study.

In the current study, analysis of physical activity showed that the odds of prediabetes were 2 fold among participants who engaged in moderate intensity work and this remained statistically significant even after controlling for other explanatory factors. Moderate intensity work was measured by participant reports on whether their activities at work increase breathing rate and heart rate for at least 10 min continuously and how many times these are done in a week.The lack of such activities was used as the referent group in the analysis. It is expected that when there are no moderate intensity work activities, the odds of prediabetes would be higher compared to if an individual has moderate intensity work. However this was not the case with our findings. Moderate intensity work activities may not provide any difference in insulin sensitivity and glucose utilization compared to low intensity activities, but studies have shown this to be the case for vigorous intensity work. In a medical hypothesis paper by Conrad Earnest, he demonstrated from various studies, that higher intensity of physical activity or work is more protective than moderate physical activity or work [21].

The study findings further show that consumption of a higher level of diet categorized as healthy was negatively associated with prediabetes. The odds of prediabetes in individuals who consumed such diet at least twice a week or more than 3 times in a week was 5 fold contrary to our expectation, and this remained statistically significant even after controlling for other explanatory variables. This finding is also in contrast to a study that found that, plant based diet was protective for prediabetes when compared to animal based diet[22]. However another study that compared fruits and vegetable consumption in prediabetic individuals demonstrated that higher intake of dark yellow vegetables was significantly associated with a higher chance of prediabetes [23].Our study findings have showed that foods traditionally considered healthy and consumed extensively by most of the people in this rural area may not be very protective in prediabetes. Most of the diet in this rural area is plant based food with high fiber and high carbohydrate content. The preparation of such foods that constitute a healthy diet with regard to prediabetes may also have a significant effect on their protective value. Most households in this rural area may not be aware of how to properly prepare such foods to retain their nutritive and protective value. The implication of our findings is that, caution need to be taken on dietary counselling and recommendations to these rural community members on diet and prevention of prediabetes or diabetes.

In the review of diet on diabetes, Riccardi et al. demonstrated that the effect of diet on prediabetes and diabetes depends on the glycemic index or glycemic load of food items [24].In a randomized pilot trial by Laura R Saslow and colleagues it was shown that very low carbohydrate ketogenic diet was more favorable in reducing the risk of prediabetes than moderate carbohydrate diets[25]. Diet recommendation as healthy for prediabetes need to go beyond just naming of food groups but also take into consideration the glycemic index, glycemic load and ketogenic effects of individual local food items.

Increasing level of BMI was independently associated with prediabetes. Obese individuals were 3 times more likely to have prediabetes. This association was also found in a study among adult teachers in urban Iran [26] and in a comparative study among adult Chinese and the Swedish [25].Several other studies have demonstrated that a high BMI is a significant risk factor for prediabetes [27] [28] [29].Our current study confirms the significance of a high BMI as an independent predictor of prediabetes. This is the first study in Uganda to document a significant association between BMI and prediabetes in a predominantly rural population. This refutes the idea that rural people are at a reduced risk of the obesity pandemic and its poor health outcomes like prediabetes, diabetes and hypertension.

Our study did not establish an independent significant association between hypertension and prediabetes. However, at the bivariate analysis the association was statistically significant. Several studies have found statistically significant association between hypertension and prediabetes [30] [31] [32] [33]. The study did not establish a significant association of hypertension and prediabetes due to low prevalence of hypertension among the prediabetic respondents. Prediabetes is among the major elements of metabolic syndrome together with hypertension and dyslipidemias. Some of the significant factors associated with prediabetes, like advancing age and BMI are also known risk factors for hypertension [34].

Whereas prediabetes may not cause an individual to seek for medical attention, people with prediabetes are at a higher risk of progressing to type 2 diabetes. Prediabetes therefore presents the best opportunity to initiate interventions for prevention of diabetes at community and at individual level.

### Limitations

One limitation to our study was the use of participants self-reports to assess physical activity and dietary patterns. Self-reports are subjective and prone to misreporting and thus may result in false associations. However care was taken to clearly define levels of physical activity and provide elaborate demonstrations of physical activity categories. While for dietary food intakes local examples in the local language were provided to minimize recall bias. Another limitation of the study was ensuring fasting status of the participants in the early morning. The study involved taking finger capillary blood samples for fasting blood glucose test early in the morning of day 2. In the rural setting and the timing of the study, participants wake up very early and eat a meal before going for work, as is their routine. Some of the participants could have forgotten and taken a meal and refuse to divulge the information because of their interest to know their blood glucose status. We ensured fasting status by cross examination every morning and making sure that the team reaches households very early to test for blood Glucose before participants leave for work or take any kind of food or drink.

## Conclusion

Prediabetes is prevalent among community members in rural Uganda. The prevalence is marginally higher but comparable to global estimates. It has been found that prediabetes affects women more than men and community members of older age especially those above 40 years. Advancing age and high body mass index are independent predictors of prediabetes in rural south western Uganda. Moderate intensity level of work was associated with prediabetes and a diet categorized as healthy was not protective as such for prediabetes.

Given the magnitude of prediabetes recognized in this rural population, it is recommended that routine screening for prediabetes should be emphasized as part of disease prevention and control services. Such screening for prediabetes should particulary target adults with a high BMI. It is further recommended that an in-depth study focusing on understanding the relationship between the commonly consumed foods and prediabetes be conducted in this rural community.

## Data Availability

The datasets analyzed during the current study are available from the corresponding author at the email; rweyeshera@gmail.com.

## Declarations

We hereby declare that this research report is the result of our original research work and inputs. No part of this report has ever been published in any journal. The research was solely undertaken as part of academic qualification requirement at Mbarara University of science and technology and all methods and procedures were done according to the relevant guidelines and regulations.
